# Leveraging diverse cell-death patterns to predict the prognosis, immunotherapy and drug sensitivity of clear cell renal cell carcinoma

**DOI:** 10.1038/s41598-023-46577-z

**Published:** 2023-11-20

**Authors:** Xi Zhang, Mingcong Zhang, Lebin Song, Shuai Wang, Xiyi Wei, Wenchuan Shao, Ninghong Song

**Affiliations:** The First Affiliated Hospital of Nanjing Medical University, Nanjing, 210029 China

**Keywords:** Biotechnology, Cancer models, Tumour biomarkers, Renal cell carcinoma

## Abstract

Clear cell renal cell carcinoma (ccRCC) poses clinical challenges due to its varied prognosis, tumor microenvironment attributes, and responses to immunotherapy. We established a novel Programmed Cell Death-related Signature (PRS) for ccRCC assessment, derived through the Least Absolute Shrinkage and Selection Operator (LASSO) regression method. We validated PRS using the E-MTAB-1980 dataset and created PCD-related clusters via non-negative matrix factorization (NMF). Our investigation included an in-depth analysis of immune infiltration scores using various algorithms. Additionally, we integrated data from the Cancer Immunome Atlas (TCIA) for ccRCC immunotherapy insights and leveraged the Genomics of Drug Sensitivity in Cancer (GDSC) database to assess drug sensitivity models. We complemented our findings with single-cell sequencing data and employed the Clinical Proteomic Tumor Analysis Consortium (CPTAC) and qRT-PCR to compare gene expression profiles between cancerous and paracancerous tissues. PRS serves as a valuable tool for prognostication, immune characterization, tumor mutation burden estimation, immunotherapy response prediction, and drug sensitivity assessment in ccRCC. We identify five genes with significant roles in cancer promotion and three genes with cancer-suppressive properties, further validated by qRT-PCR and CPTAC analyses, showcasing gene expression differences in ccRCC tissues. Our study introduces an innovative PCD model that amalgamates diverse cell death patterns to provide accurate predictions for clinical outcomes, mutational profiles, and immune characteristics in ccRCC. Our findings hold promise for advancing personalized treatment strategies in ccRCC patients.

## Introduction

Renal cell carcinoma (RCC) is the most prevalent form of kidney cancer, with a steadily increasing incidence^1,2^. Traditionally, RCC is classified into three subtypes based on morphological characteristics: clear cell, papillary, and chromophobe subtypes^3^. Clear cell renal cell carcinoma (ccRCC), accounting for 80–90% of RCC cases, is associated with the prognosis and highest mortality rate compared to the other two subtypes^4^. Despite notable advancements in the management of ccRCC, the primary treatment modality remains surgery, and adjuvant therapies have demonstrated limited effectiveness^5^. Consequently, the survival outcomes for ccRCC remain unsatisfactory, with 5-year cancer-specific survival rates reported at 75.8% in a study involving 4034 ccRCC patients across five centers in Germany^5^. In light of these limitations in current treatment strategies, there is an urgent need to explore novel therapeutic targets to enhance prognosis and patient outcomes.

Recently, attention has shifted towards a different mechanism associated with tumor formation-programmed cell death (PCD). PCD encompasses a spectrum of cell death pathways regulated by various mechanisms, including apoptosis, pyroptosis, necroptosis, ferroptosis, entotic cell death, parthanatos, netotic cell death, autophagy-dependent cell death, lysosome-dependent cell death, alkaliptosis and oxeiptosis^6^. Apoptosis, for instance, is a well-characterized process leading to cell removal without eliciting inflammatory responses, involving specific changes such as solidification, nuclear cleavage, and nucleolysis^7,8^. Necroptosis, initially regarded as alternative form of apoptosis, displays necrotic cell death morphology and concurrent autophagy activation^9,10^. Pyroptosis, on the other hand, represents an inflammatory form of PCD triggered by specific inflammasomes and the release of cytokines like IL-18 and IL-1β^11^. Ferroptosis is characterized by the accumulation of iron-dependent lipid hydroperoxides to lethal levels^12^. Entotic cell death is executed non-cell-autonomously by lysosomes and autophagy proteins^13^. Reticular cell death, reliant on the release of neutrophil extracellular traps (NETs) and reactive oxygen species (ROS) produced by NADPH oxidase, distinguishes itself from other PCD types^14,15^. Parthanatos, in contrast, does not depend on caspase mediation but instead relies on the overactivation of ribozyme, PARP-1^16^. Lysosome-dependent cell death is marked by lysosomal destabilization and a reliance on lysosomal membrane permeabilization^17^. Autophagy, the process of transporting cellular components to lysosomes for recycling, gives rise to autophagy-dependent cell death, mechanistically distinct from apoptosis or necrosis^18^. Alkaliptosis is a pH-dependent PCD form regulated by intracellular alkalinisation^19^. Lastly, oxeiptosis represents a caspase-independent and non-inflammatory form of PCD, induced by ROS and capable of triggering other PCD types, including pyroptosis, apoptosis, ferroptosis, necroptosis and autophagy-dependent cell death^20,21^. It has been established that defects in PCD are associated with cancer development, metastasis, and resistance to anticancer therapy^22^.

In tumor microenvironment (TME), the occurrence of PCD often drives a shift toward immunosuppressive TME^23^. On the one hand, the immunosuppressive conditions of low pH, hypoxia and reactive oxygen species in TME can mediate a range of programmed cell deaths in cytotoxic immune cells and promote the growth of pro-tumorigenic immune cells such as regulatory T cell (Treg), M2 macrophages and myeloid-derived suppressor cells (MDSCs)^24,25^. Tumor cells, on the other hand, tend to exhibit a loss of programmed death transduction signals and thus show an attenuated PCD state^26^. But every coin has two sides. With the occurrence of PCD, released intracellular components, including cytokines, small molecules, mitochondrial DNA, and non-coding RNA, etc. modulate the shaping of the immune landscape of the TME^27,28^. For example, IL1β, an end product from pyroptosis, can play an active role in anti-tumor immunity by signaling cascades that activate dendritic cells, macrophages, and professional antigen-presenting cells, and by regulating the Th1/Th17 differentiation of CD4^+^ T cells and CD8^+^ T cell effector function^29^. HMGB1, as a danger-associated molecular pattern molecule released by immunogenic PCD, can play a role in directly triggering the proliferation of T and B lymphocytes and down-regulating the expression of immunosuppressive CTLA4 and Foxp3 and the secretion of IL-10 in Treg cells via the TLR pathway^30–33^. Therefore, exploring the effect of PCD on TME tumor immune status in specific tumors and attempting to induce cancer cell-specific PCD or amplify the antitumor effects of PCD modulators may pave the way for scientists to inhibit and remove tumor cells without compromising antitumor immunity.

However, despite significant progress in understanding the molecular mechanisms of PCD, the clinical implications of PCD in ccRCC largely remain unclear. In this study, we aimed to bridge this knowledge gap by identifying the molecular alterations and clinical relevance of PCD-related genes in ccRCC. We have also introduced a novel indicator, the PCD-related signature (PRS), designed to predict the efficacy of therapeutic interventions and prognosis in ccRCC patients. Utilizing the PRS may offer a promising avenue for selecting more appropriate therapeutic regimens for ccRCC patients in the future.

## Materials and methods

### Data acquisition and processing

We obtained transcriptomic and clinical data on ccRCC from The Cancer Genome Atlas (TCGA) and Gene Expression Omnibus (GEO) databases. From previous literature, 1078 genes related to PCD were counted^34^. We also used the E-MTAB-1980 dataset for external validation. Additionally, we downloaded three single-cell sequencing datasets (GSE131685, GSE152938, and GSE171306) from GEO.

### Single-cell sequencing data processing

We initially had four ccRCC samples and four normal samples, totaling 64,926 cells. We processed the single-cell data using the R package Seurat. This involved: Quality Control: We filtered out low-quality cells based on criteria such as the number of expressed genes (100 < x < 6000) and the percentage of mitochondrial gene expression (< 20%). Genes with low expression (present in fewer than 100 cells) were also filtered out. This left us with 17,304 genes and 50,201 cells. Batch Effect Removal: To remove batch effects, we employed the FindIntegrationAnchors (with the reduction parameter set to “rpca”) and IntegrateData functions. Dimensionality Reduction: We reduced data dimensionality using the RunPCA and RunUMAP functions, retaining the top 30 principal components and the top 2000 highly variable genes. Clustering and Grouping: Cells were clustered and grouped using the FindNeighbors and FindClusters functions with a resolution parameter of 1.5. Cell sub-populations were defined using classical marker genes from the literature. Differentially expressed genes (DEGs) between tumor and normal cells were identified with criteria of adj. P-value < 0.001 and |logFC| > 1.

### Construction of the PCD-related signature

We screened for genes that were differentially expressed in both single-cell data and TCGA datasets. To construct the PRS, we used univariate Cox regression analysis and LASSO analysis. By linearly combining the gene expression-weighted regression coefficients, we obtained the PRS formula. The algorithm was as follows: PRS = Coef A * Gene A expression + Coef B * Gene B expression +  ⋯ + Coef N * Gene N expression, where Coef represented coefficients calculated by LASSO and Gene expression referred to the expression of PRGs. Patients were categorized into high and low PRS groups based on the median PRS. We divided patients into trainRisk and testRisk groups in a 6:4 ratio, using the E-MTAB-1980 dataset for external validation. Prognostic characteristics of PRS were assessed using time-dependent receiver operating characteristic (ROC) curves, Kaplan–Meier (KM) curves, univariate and multivariate Cox regression analyses.

### Construction of the PCD-related clusters and bioinformatics analysis

PCD-related clusters were formed based on the expression profiles of the modeled PRGs using consensus clustering. We estimated overall survival (OS) differences between clusters using the KM method. DEGs were identified using criteria of |log 2 (fold change FC)| > 2 and adjusted P value < 0.001 to further analyse the differences in biological pathways between clusters. Gene Ontology (GO) and Kyoto Encyclopedia of Genes and Genomes (KEGG) analyses were conducted on the DEGs. Gene Set Variation Analysis (GSVA) enrichment analysis was performed using the R package “GSVA” and “c2.cp.kegg.v7.4.symbols” from MSigDB to evaluate pathway enrichment.

### Immune microenvironment assessment and mutation analysis

We used multiple algorithms to assess immunoinfiltration in ccRCC samples, including ESTIMATE and tracking Tumor Immunophenotype (TIP, http://biocc.hrbmu.edu.cn/TIP/). Immunotherapy data were obtained from The Cancer Immunome Atlas (TCIA), and differences in immunotherapy (anti-PD-1 and anti-CTLA4) between groups were analyzed. Somatic variant data were presented in Mutation Annotation Format (MAF), and the “Maftools” package was used to illustrate mutational profiles of different risk levels through waterfall plots.

### RNA extraction, reverse transcription, and qRT-PCR

RNA-easy isolation reagent (Vazyme, China) was employed to extract total RNAs from cultured cells or tissues following the manufacturer’s instructions. The RNA levels were assumed by using RTIII All-in-One Mix with dsDNase and ChemoHS qPCR Mix (Monad, Wuhan, China). Gene expression was normalized to ACTIN. The relative expression of mRNAs was quantified by using the 2−∆∆Ct method. The primers used were shown in the Table S1.

### Statistical analysis

All analyses were performed by using R 4.2.2. Statistical tests were two-sided, with a P-value < 0.05 was considered significant, unless otherwise noted. We used the KM curve and log-rank test to assess the correlation between PRGs and OS in ccRCC patients. Adjustment for multiple testing was done using the Benjamini–Hochberg (BH) method for adjusted P-values or false rate discovery (FDR).

### Ethics approval

All the patients provided written informed consent, and the protocol was approved by ethical committee of The First Affiliated Hospital of Nanjing Medical University.

## Results

### Construction and validation of the PCD-related signature

**Figure 1:**
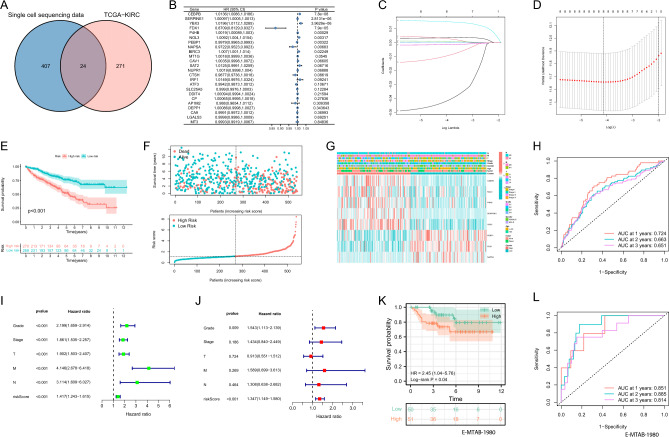
Establishment of programmed cell death related signature. (**A**) Screening of 24 different expression PRGs between single cell sequencing data and TCGA–KIRC; (**B**) Univariate COX results for 24 different expression PRGs; (**C**,**D**) Lasso analysis of prognostic PRGs with minimum lambda value; (**E**) KM curve for survival difference in PRS groups; (**F**) The risk curve of each sample reordered by PRS and the distribution of survival states. (**G**) Distribution of PRGs expression profile and clinicopathological characteristics in PRS; (**H**) ROC curves about PRS in 1, 2, 3 years. (**I**,**J**) The results of univariate and multivariate cox analysis of PRS; (**K**) KM curve for survival difference in PRS groups in E-MTAB-1980; (**L**) ROC analysis of about PRS in E-MTAB-1980.

A total of 24 differentially expressed PRGs were meticulously screened in single-cell sequencing data and TCGA, employing strict criteria (|logFC| > 1 and FDR < 0.001), and the resulting intersection was depicted in Fig. 1A. Subsequently, a comprehensive selection process via univariate COX regression analysis yielded 9 prognostic PRGs (Fig. 1B). To construct PRS for prognostication, we harnessed the power of LASSO regression analysis, which enabled us to identify 8 pivotal PRGs (Fig. 1C,D). The PRS was found to exhibit a positive correlation with patient mortality, vividly demonstrated by the PRS distribution, survival status, and KM survival curve (Fig. 1E,F). The heatmap illustrated the distribution of the 8 modeled gene expression profiles alongside clinicopathological features (Fig. 1G). Moreover, the ROC curves for PRS at 1, 2, and 3 years stood at 0.724, 0.663, and 0.651, respectively (Fig. 1H). Furthermore, univariate and multivariate Cox analyses underscored the precision of PRS in prognosticating ccRCC patients (Fig. 1I,J). To fortify the reliability of our model, we applied the E-MATB-1980 dataset for external validation, with the KM curve affirming that the high PRS group portended a less favorable prognosis (Fig. 1K). ROC curves for PRS at 1, 2, and 3 years in the E-MATB-1980 dataset yielded AUCs of 0.851, 0.885, and 0.814, respectively (Fig. 1L).

### Internal validation of the PCD-related signature

To bolster our findings, we adopted a 6:4 randomization ratio to divide patients into training and test groups for internal validation. In both groups, higher PRS values were consistently associated with poorer prognoses, as evidenced by PRS distribution, survival status, and KM curves (Fig. S1A–D). The comprehensive analysis of gene expression profiles and clinicopathological features was summarized in Fig. S1E,F. ROC analysis exhibited the remarkable prognostic value of PRS in both the training (AUC = 0.751) and test (AUC = 0.680) sets (Fig. S1G,H). Furthermore, univariate and multivariate Cox regression analyses confirmed the independent predictive role of PRS in both training and test groups (Fig. S1I–L). The meticulous validation process underscored the stability and robustness of PRS as a prognostic predictor.

### Identification of immune characteristics of the PCD-related signature

**Figure 2:**
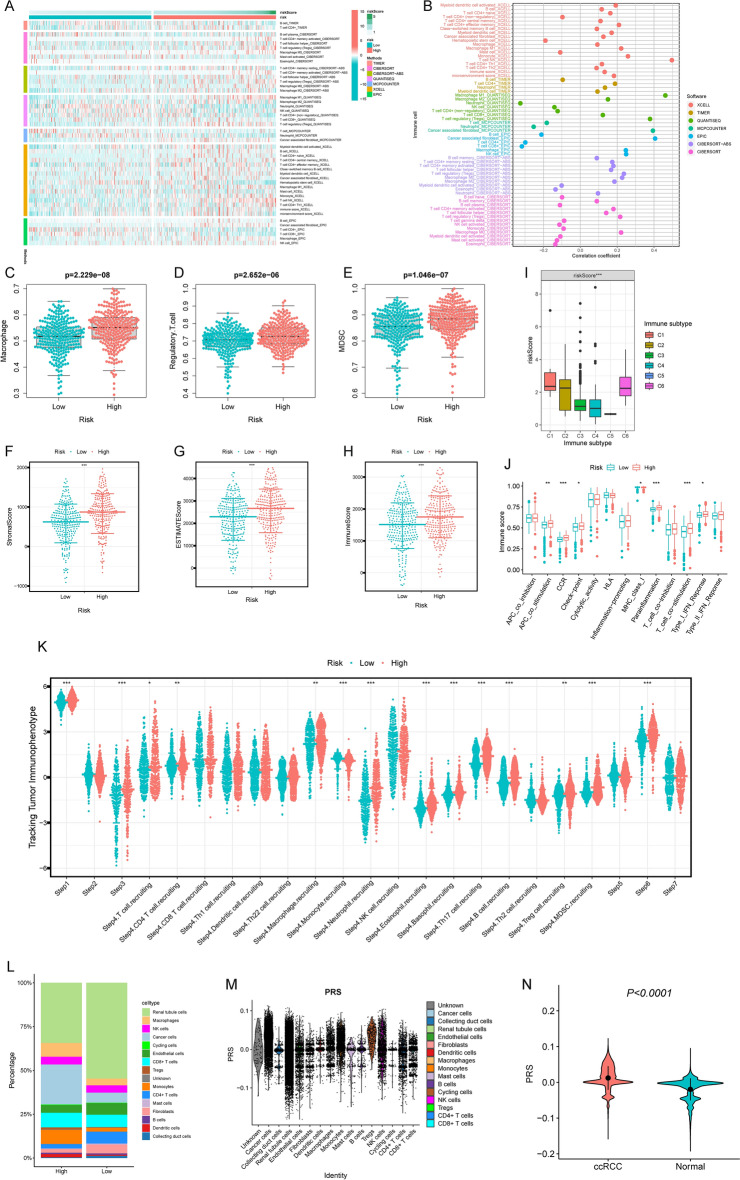
The immune characteristics of Programmed cell death signature. (**A**) Heatmap representing expression of immune cells in programmed cell death related signature groups under various algorithms; (**B**) Correlation analysis between immune cells and programmed cell death related signature under various algorithms; (**C**–**E**) Expression difference of major immunosuppressive infiltrating cells (MDSCs, macrophages, and Tregs) in PRS groups; (**F**–**H**) Differences in tumor microenvironment scores in PRS groups; (**I**) Differential expression of PRS in immune subtypes; (**J**) Differential expression of immune molecular functions in PRS groups; (**K**) Differential expression of PRS in different Tracking Tumor immunophenotypes; (**L**) Proportions of different cells between the high and low PRS groups; (**M**) The distribution of PRS in various immune cells and tumor cells; (**N**) PRS was significantly up-regulated in ccRCC tumors tissues compared with normal samples.

Employing seven different algorithms, we generated a heatmap illustrating the diverse immune cell components (Fig. 2A). The relationship between PRS and immune cells, as delineated by various algorithms, was displayed in Fig. 2B. Furthermore, we observed significantly higher expression of three immunosuppressive cells in the high PRS group (Fig. 2C–E). Utilizing the ESTIMATE algorithm, we discerned that immune score, stromal score, and ESTIMATE score were all elevated in the high PRS group (Fig. 2F–H). To corroborate the validity of PRS for immunotyping, we investigated the association between PRS and immune subtypes, revealing differential expression in subtypes C1, C6, C3, C4, and C5 (Fig. 2I). Moreover, the high PRS group exhibited markedly elevated levels of immune-related molecules in comparison to the low PRS group (Fig. 2J). In our quest to delve deeper into the role of immune cells in ccRCC progression, we evaluated immune activity scores at each step using data from the TIP database. Impressively, we found that the high PRS group displayed significantly higher frequencies of anti-tumour immune cells (Fig. 2K). Our exploration further extended to the spatial distribution of PRS within ccRCC tissues using single-cell signature scoring. Strikingly, we embarked on a detailed exploration of the spatial distribution of PRS within ccRCC tissues. The high PRS group exhibited a substantial increase in the proportion of cancer cells, macrophages, and Treg cells (Fig. 2L), and notably, higher PRS expression within these cell types (Fig. 2M). Additionally, we detected a substantial enrichment of PRS in ccRCC tumors compared to normal tissue samples, as determined by the “AddModuleScore” algorithm (P < 0.0001; Fig. 2N).

### Drug susceptibility analysis of the PCD-related signature

**Figure 3:**
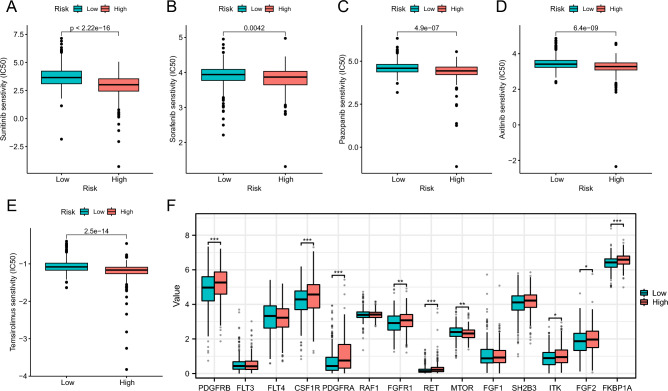
Drug sensitivity and target analysis of the programmed cell death related signature. Sensitivity analysis for Sunitinib (**A**), Sorafenib (**B**), Pazopanib (**C**), Axitinib (**D**) and Temsirolimus (**E**) in PRS groups; (**F**) Differential expression of target genes in PRS groups.

To assess the susceptibility of patients with high and low PRS, we employed the “pRRophetic” package to analyze the IC50 values of five common anti-renal cancer drugs, namely Sunitinib, Sorafenib, Pazopanib, Axitinib, and Temsirolimus. Strikingly, our results demonstrated a significant overexpression of IC50 values for all five drugs in the low PRS group (Fig. 3A–E). The target genes of these anticancer drugs, obtained from the DrugBank database, encompassed PDGFRB, FLT3, FLT4, CSF1R, PDGFRA, RAF1, FGFR1, RET, MTOR, FGF1, SH2B3, ITK, FGF2, and FKBP1A. Notably, the high PRS group exhibited higher expression levels for most of these target genes (Fig. 3F). These findings underscored the potential of PRS in aiding the selection of appropriate treatment strategies, ultimately contributing to improved prognosis.

### Correlation of the PCD-related signature with clinicopathological characteristics

Within the TCGA cohort, we embarked on an exploration of the signature's applicability across diverse clinical subgroups. Our analysis revealed a significant elevation of PRS in advanced-stage cases, high-grade cases, advanced T-stage cases, positive lymph node metastasis cases, and positive distant metastasis cases (Fig. S2A–E). Similarly, patients with advanced ccRCC (grade 3 or 4, stage III or IV, T3 or T4, M1, and N1) exhibited a higher propensity to belong to the high PRS group (Fig. S2F–J). The high-PRS group consistently exhibited a poor prognosis, as depicted in Fig. S2K–T. These observations affirmed the robust predictive capability of our model across distinct clinicopathological stages.

### Identification of PCD-related clusters and bioinformatics analysis

**Figure 4:**
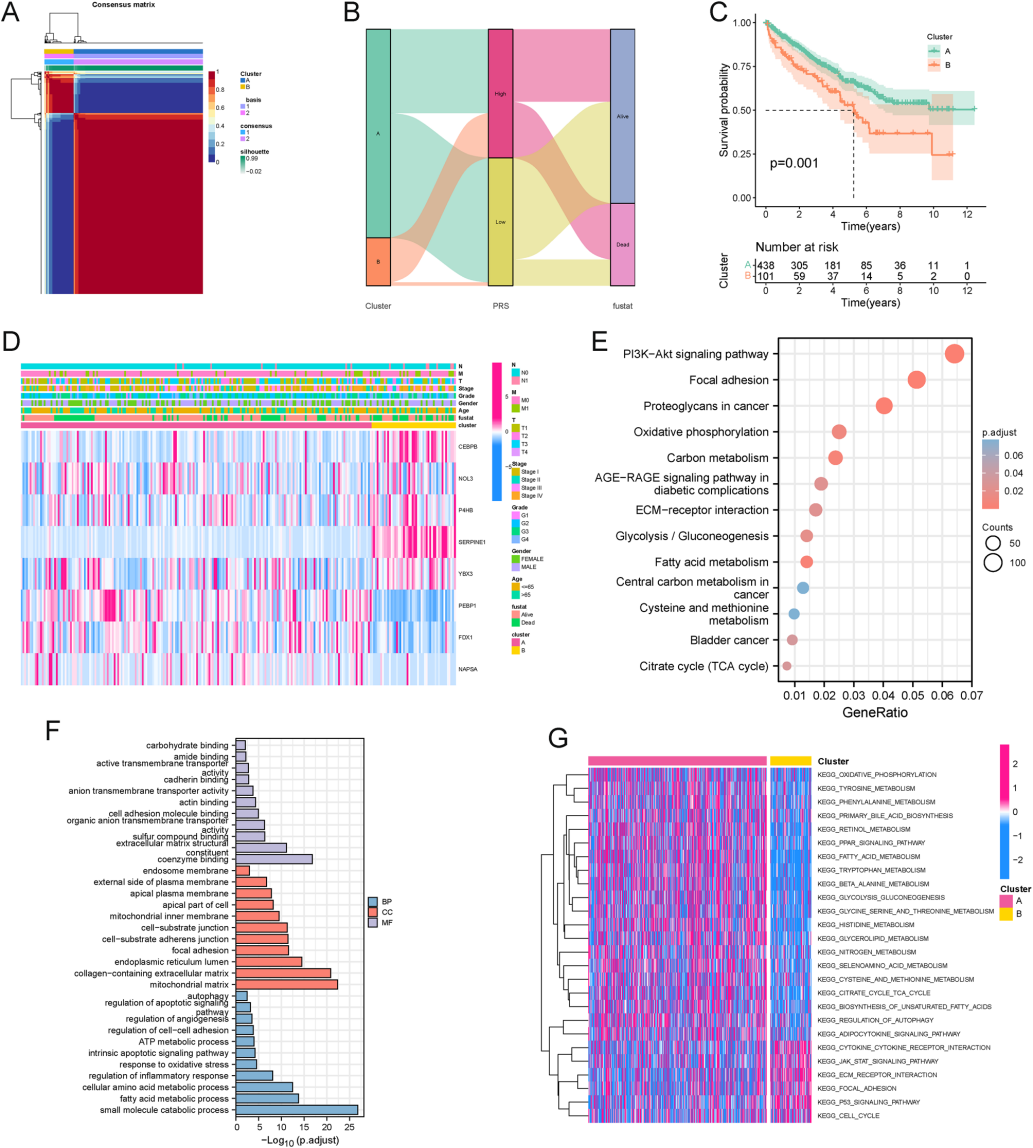
Identification and comparison of programmed cell death clusters of ccRCC. (**A**) Heatmap plot indicating the consensus matrix of NMF clustering results utilizing the gene expression profile in TCGA KIRC cohort, colored by two ccRCC clusters; (**B**) The Sankey diagram showing the correlation among programmed cell death clusters, PRS, and fustat; (**C**) Overall survival difference between cluster A and B; (**D**) Heatmap showed the distribution of clinicopathological characteristics and PRGs expression; (**E**) KEGG pathway enrichment of DEGs between clusters A and B. (**F**) GO functional annotation analysis of DEGs between clusters A and B; (**G**) Representative enriched GSVA terms between clusters.

By leveraging the expression profiles of the modeling genes, we categorized patients into two clusters using the NMF algorithm (Fig. 4A). The KM survival curve clearly delineated a worse prognosis for cluster B in comparison to cluster A (Fig. 4B). The Sankey diagram further illustrated the relationship between classification, PRS, and survival status, indicating that a majority of cluster B patients belonged to the high PRS group, with a notable concentration of deaths therein (Fig. 4C). This observation was consistent with our previous analyses. The heatmap provided a visual representation of the distribution of the 8 modeling gene expression profiles alongside clinicopathological characteristics (Fig. 4D). In an effort to explore the heterogeneity within each PCD-related cluster, we conducted functional enrichment analysis on the DEGs between the clusters. KEGG and GO analysis highlighted significant enrichment in pathways such as the PI3K-Akt signaling pathway, various metabolic pathways, mitochondrial matrix regulation, inflammatory response regulation, and angiogenesis regulation (Fig. 4E,F). Additionally, GSVA revealed significant enrichment of cancer-promoting pathways, including the P53 signaling pathway, JAK-STAT signaling pathway, cell cycle, and ECM receptor interaction, within cluster B (Fig. 4G).

### Mutation and immunotherapeutic responses of the PCD-related signature

**Figure 5:**
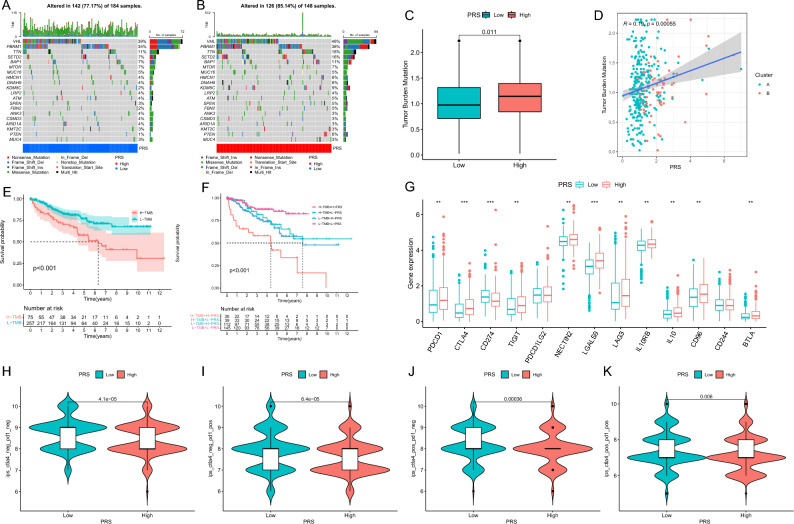
Mutational and immunotherapeutic characteristic of programmed cell death signature. (**A**,**B**) Waterfall plots of somatic mutations in tumors in PRS groups; (**C**) Differential expression of tumor burden mutation (TMB) in PRS groups; (**D**) Correlation analysis among tumor burden mutation, cluster and PRS; (**E**) KM curve for survival difference in TMB groups; (**F**) KM curves for the survival difference for combination of TMB and programmed cell death related signature; (**G**) Differential expression of immunosuppressive checkpoints in PRS groups; (**H**–**K**) Differential expression of anti-CTLA4 and/or anti-PD1 combination immunotherapy in PRS groups.

Given the significant correlation between Tumor Mutation Burden (TMB) and immunotherapy efficacy, we explored the link between PRS and TMB. Interestingly, the mutation rate was 77.17% in the low PRS group and 85.14% in the high PRS group, with TMB being significantly higher in the latter (Fig. 5A–C). A positive correlation between PRS and TMB was evident, with cluster B exhibiting higher PRS and TMB than cluster A (Fig. 5D). The KM curve indicated a poor prognosis in the high TMB group (Fig. 5E). Furthermore, combining PRS and TMB for prognosis prediction revealed that H-TMB + H-PRS had the worst prognosis, while L-TMB + L-PRS had the best prognosis (Fig. 5F). Then, previous studies have shown that TMB has an association with the outcome of immunotherapy. Our analysis of immunosuppressive checkpoint expression confirmed significant overexpression in the high PRS group (Fig. 5G). Additionally, the low PRS group displayed a higher probability of responding to CTLA4, PD-1, and CTLA4 + PD-1 immunotherapy, further emphasizing the clinical relevance of PRS (Fig. 5H–K).

### Correlation of the modeling genes with clinicopathological characteristics

In Fig. S3A, we observed differential expression of the 8 modeling genes between cancer and normal tissues, with 5 genes up-regulated in cancer tissue and 3 down-regulated. Notably, all 8 modeling genes demonstrated substantial predictive capacity based on the AUC (Fig. S3B). Differential expression of these genes across various clinicopathological stages was highlighted in Fig. S3C–G. Patients were stratified into high-expression and low-expression groups based on median gene expression, revealing that 5 genes were positively correlated with poor prognosis, while 3 genes were inversely correlated (Fig. S3O).

### Single-cell transcriptomic context of the 8 modeling genes

For an in-depth exploration of the potential mechanisms governed by the modeling genes in ccRCC, we leveraged three single-cell sequencing datasets from the GEO database (GSE131685, GSE152938, and GSE171306). After meticulous quality control, we analyzed 17,304 genes across 50,021 cells obtained from four ccRCC and four normal samples (Fig. S4A). Subsequently, 16 distinct clusters and 15 cell types were identified, encompassing immune cells, tubular cells, endothelial cells, and tumor cells (Fig. S4B,C).

**Figure 6:**
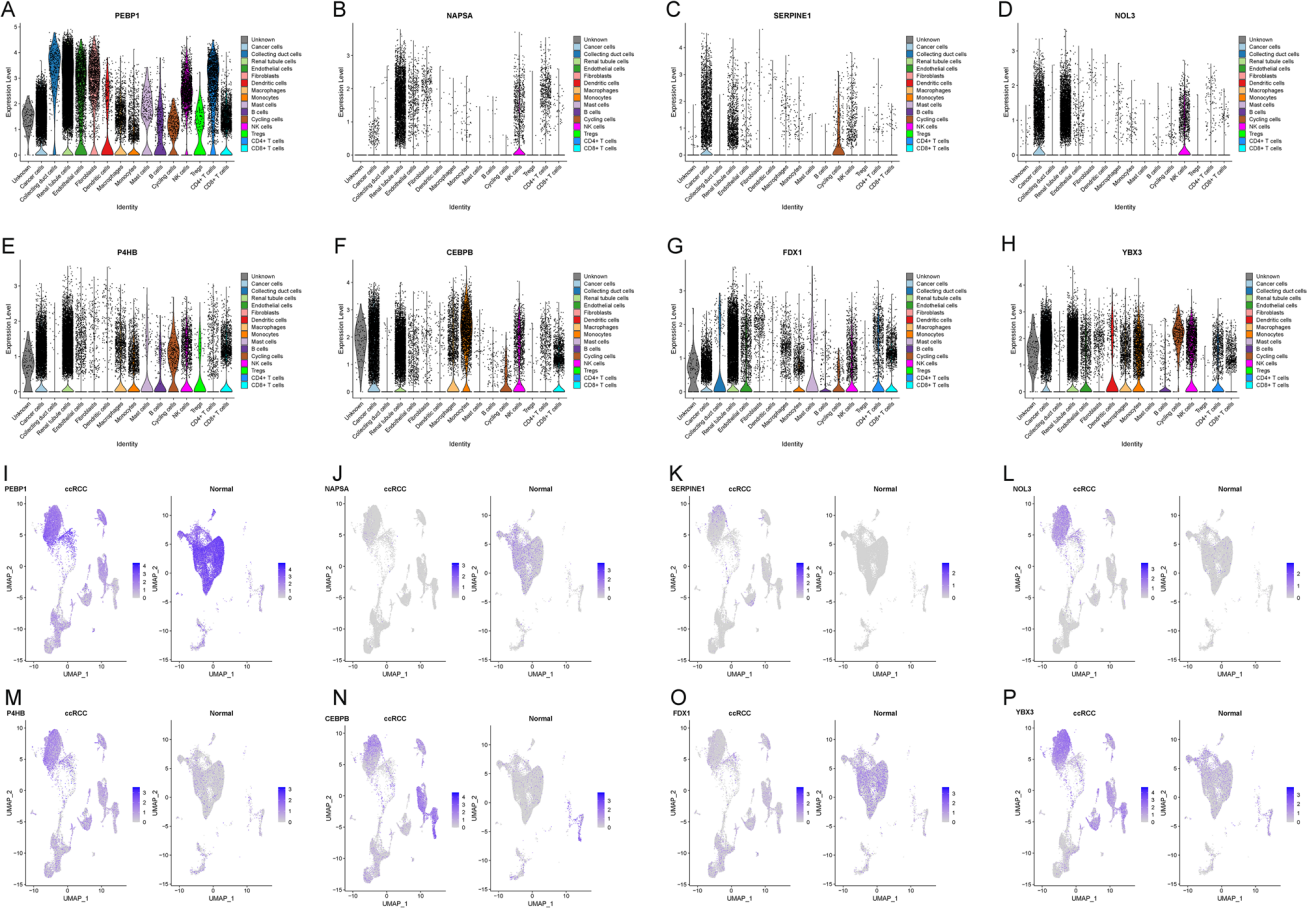
Single cell sequencing analysis of 8 modeling genes. (**A**–**H**) The distribution of 8 modeling genes in various immune cells and tumor cells; (**I**–**P**) Expression distribution of 8 modeling genes in ccRCC and normal cells.

In agreement with previous studies^35^, renal tubular epithelial cells predominated in normal renal cortical samples, while immune cells and tumor cells dominated in ccRCC, signifying the ccRCC tumor immune microenvironment (Fig. S4D). The majority of immune cells were identified in ccRCC patients, delineating the tumour immune microenvironment of ccRCC (Fig. S4E). Importantly, we examined the expression profiles of the eight modeled genes within different cell types (Fig. 6A–H) and discerned distinct distribution patterns in cancerous and paracancerous tissues. Specifically, PEBP1, NAPSA, and FDX1 exhibited higher expression in paracancerous tissues, while SERPINE1, NOL3, CEBPB, P4HB, and YBX1 were more highly expressed in cancerous tissues, reinforcing our previous analyses (Fig. 6I–P).

### Validation of protein and mRNA expression of PCD-related genes in ccRCC

**Figure 7:**
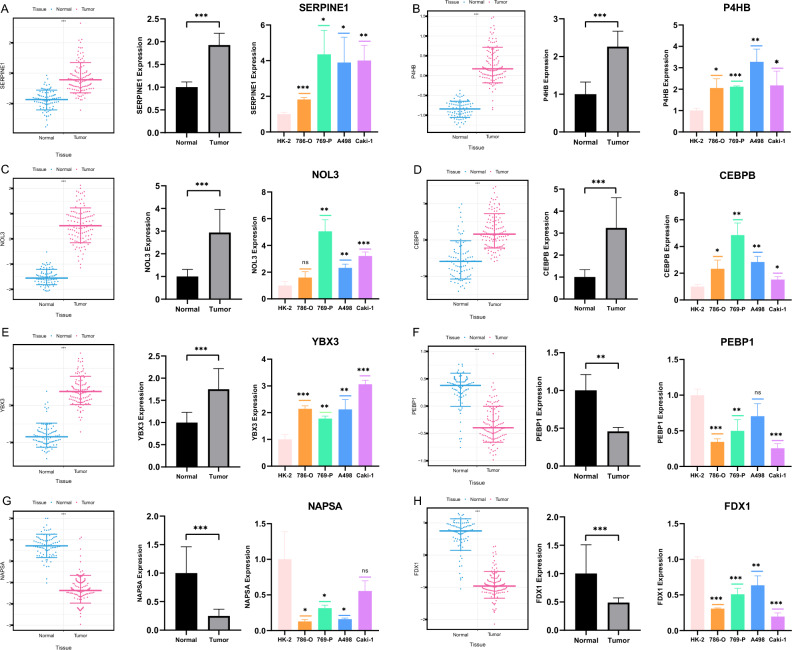
Protein and mRNA expression of the 8 modeling genes. (**A**–**H**) Differential protein and mRNA expression of 8 modeling genes in tissues and cell lines. [(**A**) SERPINE1; (**B**) P4HB; (**C**) NOL3; (**D**) CEBPB; (**E**) YBX3; (**F**) PEBP1; (**G**) NAPSA; (**H**) FDX1].

To validate the expression of PCD-related genes in ccRCC, we obtained protein expression data for the eight modeling genes from the Clinical Proteomic Tumor Analysis Consortium (CPTAC). Additionally, we performed qRT-PCR to assess differences in mRNA expression of these eight modeling genes in both tissues and cell lines. Figure 7A–E illustrated the protein and mRNA expression levels of five modeling genes (SERPINE1, P4HB, NOL3, CEBPB, and YBX3) were significantly that exhibited significant overexpression in ccRCC tissues and cell lines. In contrast, the remaining three genes (PEBP1, NAPSA, and FDX1) displayed noteworthy underexpression in ccRCC tumors, as depicted in Fig. 7F–H.

## Discussion

Traditionally, the prognosis of ccRCC has primarily relied on histological grade, tumour-node-metastasis (TNM) stage, and clinical stage. Despite incremental improvements in these indicators over the past few decades, they still exhibit limitations in accurately predicting patient OS and guiding anti-cancer treatment decisions. There is an urgent need for new biomarkers that can enhance the diagnosis and prognosis of ccRCC.

PCD plays a pivotal role in intricate regulatory processes, involving diverse mechanisms, and is of utmost importance in tumor initiation and metastasis^36^. In our study, we identified a signature comprised of eight PCD-associated genes (SERPINE1, CEBPB, NOL3, P4HB, YBX3, PEBP1, FDX1, and NAPSA). This signature demonstrated a remarkable ability to accurately predict the overall survival of ccRCC patients. The validation of our modeling genes' expression patterns in both protein and mRNA levels strengthens the credibility of our findings. The overexpression of certain genes, such as SERPINE1, P4HB, NOL3, CEBPB, and YBX3, in ccRCC tumors suggests their potential involvement in the disease pathogenesis. These findings align with previous reports implicating these genes in cancer progression and aggressiveness. Conversely, the underexpression of genes like PEBP1, NAPSA, and FDX1 in ccRCC may indicate their roles as tumor suppressors or regulators of critical cellular processes. These genes warrant further investigation to elucidate their functions in ccRCC development and progression. The validation of these gene expression patterns adds valuable insights into the molecular landscape of ccRCC. These findings could potentially serve as biomarkers for diagnosis, prognosis, or therapeutic targeting in ccRCC. Indeed, the expression patterns and roles of these PCD-related genes align with their involvement in various cancers, corroborating our findings in ccRCC: SERPINE1, known for inhibiting tissue plasminogen activators and urokinase, has been consistently found to be highly expressed in multiple cancers, such as colorectal, gastric, and lung cancers. Its association with poor prognosis is in line with our observations^37–39^. Elevated expression of CEBPB has been identified in breast cancer, colorectal cancer, and glioma. Studies have suggested its involvement in promoting the growth and metastasis of breast and colorectal cancers through aerobic glycolysis. Our results further supported its role as a pro-cancer factor in ccRCC^40–42^. NOL3 encodes an anti-apoptotic protein involved in autophagy and apoptosis regulation. High NOL3 expression has been linked to poor prognosis in colorectal cancer patients, consistent with its potential role in promoting cancer progression^43^. As a molecular chaperone, P4HB aids in responding to endoplasmic reticulum stress by improving the handling of misfolded proteins. It has emerged as a diagnostic and prognostic marker in various cancers, including gastric, bladder, and colorectal cancers. Its overexpression is associated with tumor progression, which aligns with our findings^44,45^. A member of the transcription factor family primarily expressed in endothelial cells, YBX3 plays a crucial role in regulating cell proliferation, differentiation, and tight junction protein expression. It has been implicated in promoting the proliferation and progression of liver, breast, melanoma, and bladder cancers^46,47^. Our results were in agreement with its role in cancer development.

Serving as a physiological endogenous inhibitor of the RAF1/MEK/ERK pathway, PEBP1 may inhibit cancer cell migration, proliferation, and invasion. Downregulation of PEBP1 has been observed in liver and pancreatic cancer, where it may contribute to aggressive tumor behavior and poor prognosis^48,49^. FDX1 is involved in regulating the cuproptosis pathway and copper ion carrier-induced cell death. It tends to be downregulated in most cancer types and has been associated with better outcomes, supporting our observations^50,51^. Although less extensively studied, NAPSA is considered a surface-active metabolism-related gene related to the prognosis of certain cancers, such as primary LUAD and LUAD brain metastases^52^. These collective findings underscore the significance of these PCD-related genes in cancer biology and their potential as valuable biomarkers in different cancer types, including ccRCC.

Tumor-infiltrating immune cells are known to play a pivotal role in regulating cancer cell behavior within the tumour microenvironment (TME). They exhibit significant plasticity and can exert either anti-tumour or pro-tumour functions^53^. Previous research, along with our own analysis, has highlighted the crucial role of PCD in modulating the immunosuppressive TME^23^. The presence of PCD in the TME is often accompanied by the release of intracellular components, including cytokines, mitochondrial DNA (mtDNA), and exosomes, which have a profound impact on shaping the immune landscape of the TME^27,54^. These components can either enhance the presence of anti-tumour immune cells (such as cytotoxic T cells and natural killer cells) or regulate immunosuppressive cell populations (such as regulatory T cells, myeloid-derived suppressor cells, and macrophages), ultimately influencing tumor regression or progression^55,56^. For instance, interleukin-1 (IL-1) has been shown to induce chronic inflammation, promoting tumor progression by stimulating processes like epithelial-to-mesenchymal transition, cancer cell proliferation, and the enrichment of immunosuppressive cell populations within the TME^57^. Similarly, extracellular ATP can be converted into the immunosuppressive metabolite adenosine, inducing the proliferation of tumor-infiltrating macrophages through the action of cell membrane exonucleotides CD39 and CD73^58^.

In our analysis, we observed a significant upregulation of immunosuppressive cells and immunosuppressive checkpoints in the high PRS group. PRS was notably higher in immunoassay C6 and lower in immunoassay C3. Previous studies have indicated that the C3 subtype was associated with better prognosis, while the C6 subtype was linked to worse outcomes. Consequently, it is reasonable to speculate that there exists a complex interplay between PCD, tumor immunity, and ccRCC. However, further research is needed to unravel the precise mechanisms underlying this interaction.

The strength of our study lies in its robust statistical analysis of PCD-related genes using high-throughput data and large databases, addressing the pressing need for validation indicators in ccRCC. Additionally, our research contributed to a better understanding of how PCD functions in the context of ccRCC. Nonetheless, there were some limitations to our study. First, we employed traditional univariate and Lasso regression risk analyses to construct and assess a PCD-related risk prognostic model. While these methods are well-established and widely used, future research may benefit from more advanced techniques for further refinement. Second, the clinical information available in the TCGA database was not comprehensive, and additional parameters such as CT images were not accessible for model validation.

Our study on ccRCC and its association with PCD-related genes highlights several promising avenues for future research. First, further functional validation is needed to elucidate the precise roles of these genes in ccRCC development. Second, there is potential for innovative therapeutic interventions targeting these genes or related pathways, both in preclinical and clinical settings. Third, given the link between the signature and immune infiltration, exploring immune modulation strategies, such as immunotherapies, is a compelling direction. Finally, the identified signature holds promise for the development of robust biomarkers for ccRCC diagnosis, prognosis, and treatment response prediction, with the integration of multi-omics data and liquid biopsy approaches enhancing these efforts.

## Conclusion

In summary, our study has culminated in the development and validation of an innovative 8-gene PCD-related signature for predicting the prognosis of ccRCC patients. The 8-gene PCD-related signature demonstrated robust prognostic capabilities, enabling the assessment of clinical outcomes in ccRCC patients. By leveraging this signature, clinicians can better tailor treatment strategies and provide more personalized care to individuals affected by this challenging disease. Beyond its prognostic utility, our signature is closely associated with immune infiltration patterns within ccRCC tumors. This finding underscores the potential interplay between PCD-related genes and the tumor microenvironment, shedding light on the complex immune dynamics at play in ccRCC. Overall, our study not only offers a valuable prognostic tool but also presents exciting opportunities to improve patient outcomes and advance our understanding of this complex disease by connecting PCD-related genes with ccRCC.

### Supplementary Information

Supplementary Figure S1.

Supplementary Figure S2.

Supplementary Figure S3.

Supplementary Figure S4.

Supplementary Legends.

Supplementary Table S1.

## Data Availability

The data that support the findings of this study are openly available in TCGA and GEO datasets.
