# Supplementary material for: Leveraging diverse cell-death patterns to predict the prognosis, immunotherapy and drug sensitivity of clear cell renal cell carcinoma
Source: Sci Rep. 2023 Nov 20;13:20266. doi: 10.1038/s41598-023-46577-z (PMC10662159; doi:10.1038/s41598-023-46577-z)
Supplement: Supplementary file 1 — Supplementary Figure S1. [file 41598_2023_46577_MOESM1_ESM.pdf]

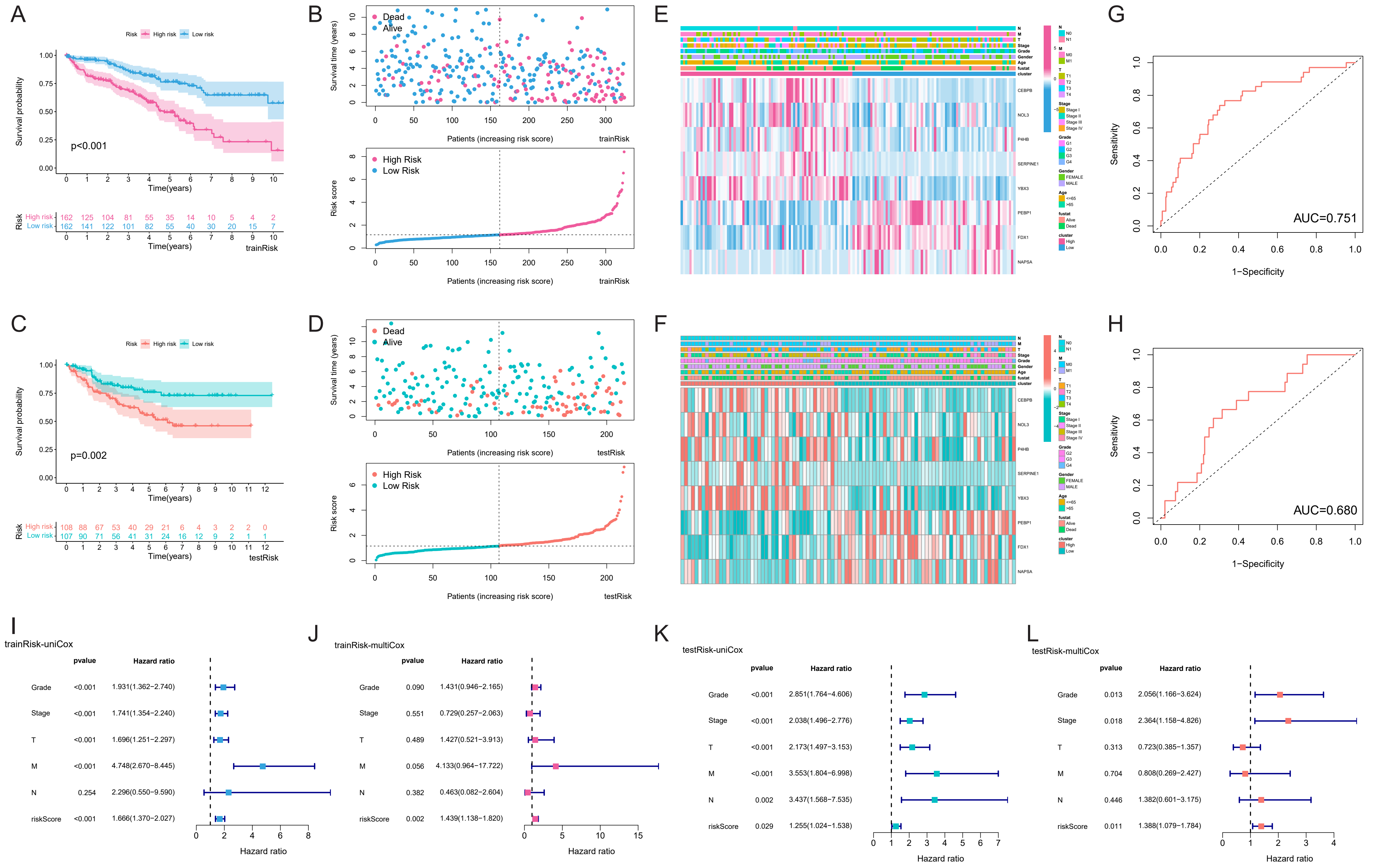

Figure S1 Internal verification of programmed cell death related signature
(A and C) KM curves for survival difference in training and test groups;
(B and D) The risk curve of each sample reordered by programmed cell death related signature and the distribution of survival states in training and test groups;
(E and F) The distribution of PRGs expression profile and clinicopathological characteristics in training and test groups;
(G and H) ROC analysis of programmed cell death related signature in training and test groups;
(I and J) The results of univariate and multivariate cox analysis of programmed cell death related signature in the training group;
(K and L) The results of univariate and multivariate cox analysis of programmed cell death related signature in the test group.
