# Supplementary material for: Leveraging diverse cell-death patterns to predict the prognosis, immunotherapy and drug sensitivity of clear cell renal cell carcinoma
Source: Sci Rep. 2023 Nov 20;13:20266. doi: 10.1038/s41598-023-46577-z (PMC10662159; doi:10.1038/s41598-023-46577-z)
Supplement: Supplementary file 2 — Supplementary Figure S2. [file 41598_2023_46577_MOESM2_ESM.pdf]

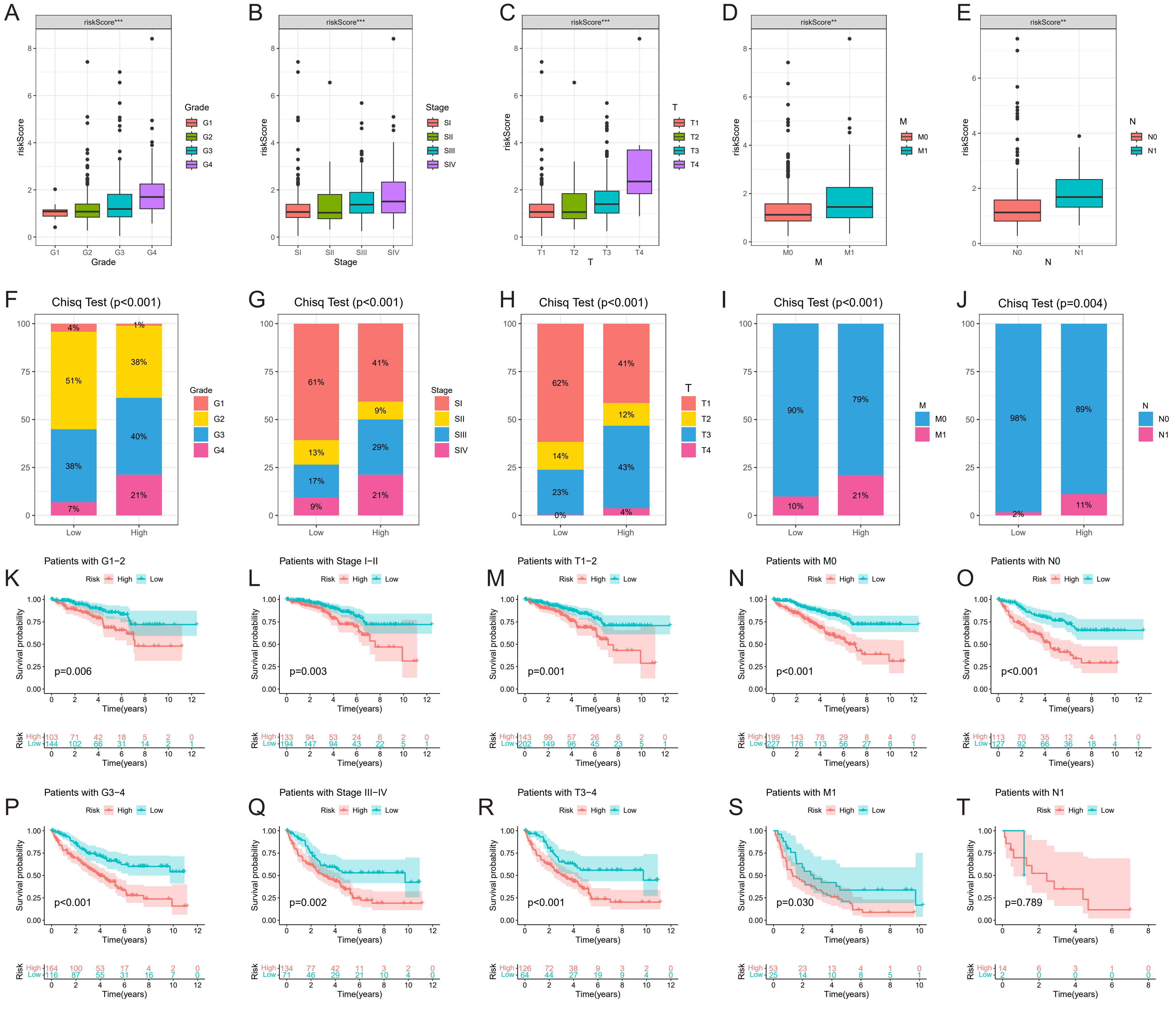

Figure S2 The correlation between programmed cell death related signature with clinicopathological features
(A-E) Different expression of the PRS among different clinicopathological subgroups;
(F-J) Difference in the proportion of different grades, stages, TMN stage in PRS groups;
(K-T) Survival analysis of programmed cell death related signature in different clinicopathological variables.
