# Supplementary material for: Leveraging diverse cell-death patterns to predict the prognosis, immunotherapy and drug sensitivity of clear cell renal cell carcinoma
Source: Sci Rep. 2023 Nov 20;13:20266. doi: 10.1038/s41598-023-46577-z (PMC10662159; doi:10.1038/s41598-023-46577-z)
Supplement: Supplementary file 3 — Supplementary Figure S3. [file 41598_2023_46577_MOESM3_ESM.pdf]

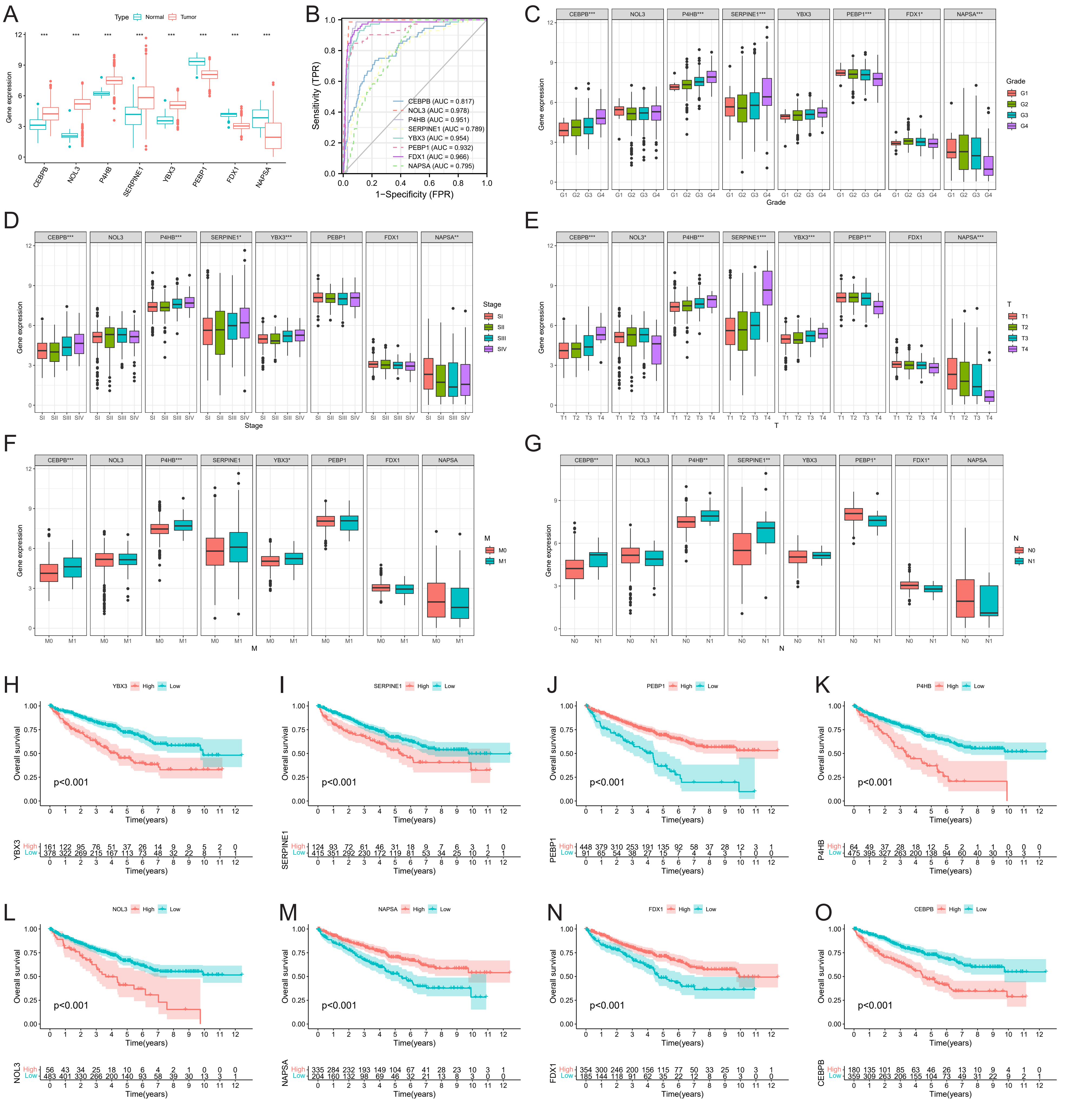

Figure S3 Identification of clinicopathological characteristics of 8 modeled genes
(A)Differential expression of 8 modeled genes between cancer and normal tissues;
(B) Time-dependent ROC analysis of 8 modeled genes;
(C-G) Differential expression of modeled genes in various clinicopathological stages (Grade, Stage, TMN stage);
(H-O) Prognostic characteristics of modeled genes.
