# Supplementary material for: Leveraging diverse cell-death patterns to predict the prognosis, immunotherapy and drug sensitivity of clear cell renal cell carcinoma
Source: Sci Rep. 2023 Nov 20;13:20266. doi: 10.1038/s41598-023-46577-z (PMC10662159; doi:10.1038/s41598-023-46577-z)
Supplement: Supplementary file 4 — Supplementary Figure S4. [file 41598_2023_46577_MOESM4_ESM.pdf]

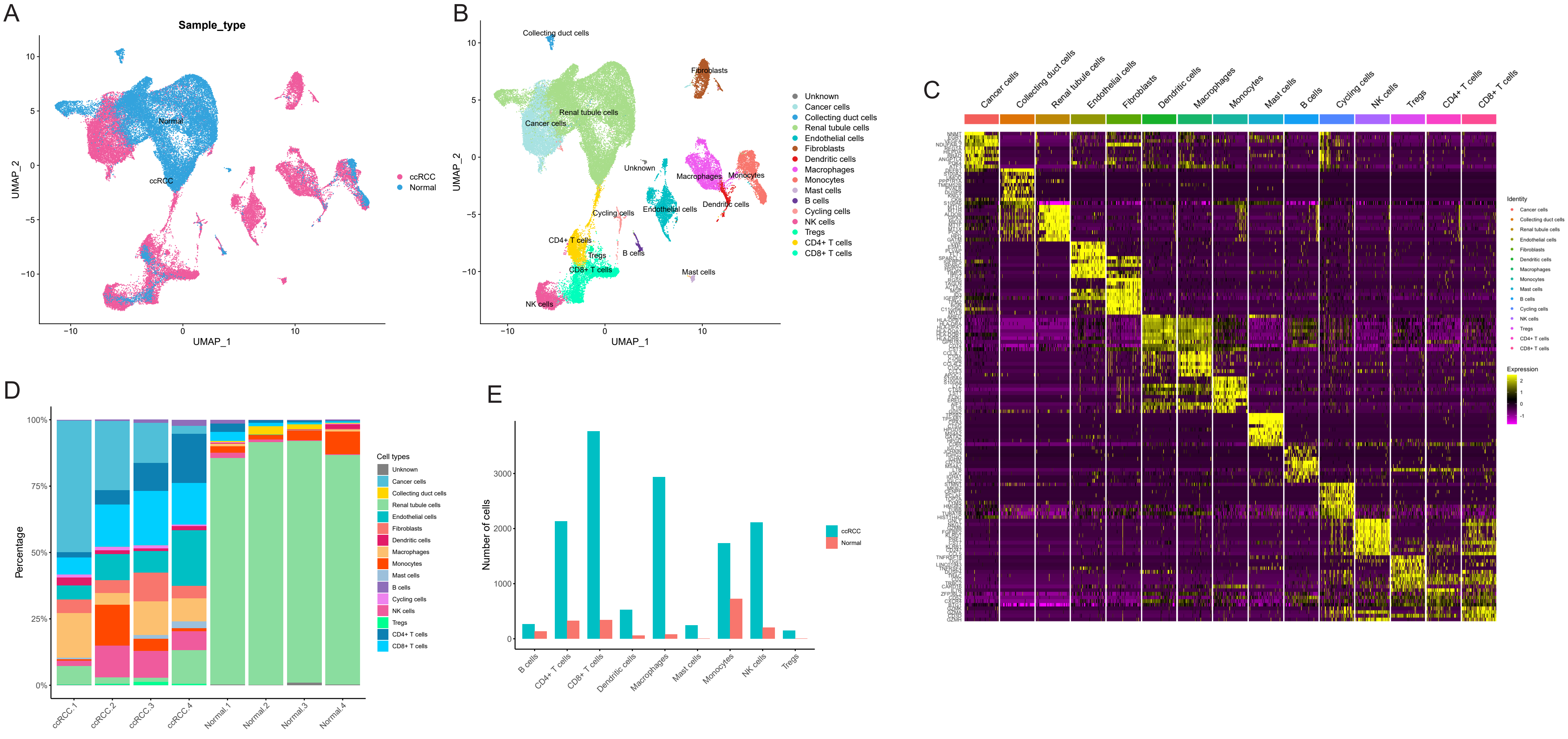

Figure S4 Cell type distribution in single cell sequencing data
(A)Composition and distribution of single cells from GSE131685, GSE152938, and GSE171306;
(B) UMAP embedding of 50201 single cells from 4 human normal kidney and 4 ccRCC samples. Labels refer to 16 clusters identified;
(C) Proportional gene expression of the top 10 specific genes in each cluster. Each column is the mean expression of all cells in a cluster;
(D) Composition and distribution of distinct cells in 8 single-cell samples;
(E) Distribution of tumor cells and normal cells in each type of immune cell.
