# Supplementary material for: Leveraging diverse cell-death patterns to predict the prognosis, immunotherapy and drug sensitivity of clear cell renal cell carcinoma
Source: Sci Rep. 2023 Nov 20;13:20266. doi: 10.1038/s41598-023-46577-z (PMC10662159; doi:10.1038/s41598-023-46577-z)
Supplement: Supplementary file 5 — Supplementary Legends. [file 41598_2023_46577_MOESM5_ESM.docx]

**Legends of supplementary information**

Figure S1 Internal verification of programmed cell death related signature

(A and C) KM curves for survival difference in training and test groups; (B and D) The risk curve of each sample reordered by programmed cell death related signature and the distribution of survival states in training and test groups; (E and F) The distribution of PRGs expression profile and clinicopathological characteristics in training and test groups; (G and H) ROC analysis of programmed cell death related signature in training and test groups; (I and J) The results of univariate and multivariate cox analysis of programmed cell death related signature in the training group; (K and L) The results of univariate and multivariate cox analysis of programmed cell death related signature in the test group.

Figure S2 The correlation between programmed cell death related signature with clinicopathological features

1. E) Different expression of the PRS among different clinicopathological subgroups; (F-J) Difference in the proportion of different grades, stages, TMN stage in PRS groups; (K-T) Survival analysis of programmed cell death related signature in different clinicopathological variables.

Figure S3 Identification of clinicopathological characteristics of 8 modeled genes

1. Differential expression of 8 modeled genes between cancer and normal tissues; (B) Time-dependent ROC analysis of 8 modeled genes; (C-G) Differential expression of modeled genes in various clinicopathological stages (Grade, Stage, TMN stage); (H-O) Prognostic characteristics of modeled genes.

Figure S4 Cell type distribution in single cell sequencing data

1. Composition and distribution of single cells from GSE131685, GSE152938, and GSE171306; (B) UMAP embedding of 50201 single cells from 4 human normal kidney and 4 ccRCC samples. Labels refer to 16 clusters identified: (C) Proportional gene expression of the top 10 specific genes in each cluster. Each column is the mean expression of all cells in a cluster; (D) Composition and distribution of distinct cells in 8 single-cell samples; (E) Distribution of tumor cells and normal cells in each type of immune cell.
