# Supplementary material for: Leveraging diverse cell-death patterns to predict the prognosis, immunotherapy and drug sensitivity of clear cell renal cell carcinoma
Source: Sci Rep. 2023 Nov 20;13:20266. doi: 10.1038/s41598-023-46577-z (PMC10662159; doi:10.1038/s41598-023-46577-z)
Supplement: Supplementary file 6 — Supplementary Table S1. [file 41598_2023_46577_MOESM6_ESM.docx]

Table S1 The list of primer sequences used in our study.

| Primer | Forward | Reverse |
| --- | --- | --- |
| CEBPB | 5′– AACTTTGGCACTGGGGCACTTG−3′ | 5′–GGGCAGAGGGAGAAGCAGAGAG−3′ |
| FDX1 | 5ʹ-CTTTGGTGCATGTGAGGGAA-3ʹ | 5ʹ-GCATCAGCCACTGTTTCAGG-3ʹ |
| NAPSA | 5’-CTTCAGTGTGCCCTGCTGGTTAC-3’ | 5’-CATCTACCCGCCCAGTTCCATATTG-3’ |
| NOL3 | 5′-AGTTCGAAGAAATGGGCAAC-3′ | 5′-AGTTCGAAGAAATGGGCAAC-3′ |
| P4HB | 5′-AGGCTGATGACATCGTGAACT-3′ | 5′-GGTATTTGGAGAACACGTCACTG-3′ |
| PEBP1 | 5′-CTCCGATTATGTGGGCTCGG-3′ | 5′-GGTGGTCTCCAGATCGGTTG-3′ |
| SERPINE1 | 5’-GCAAGGCACCTCTGAGAACT-3’ | 5’-GCAAGGCACCTCTGAGAACT-3’ |
| YBX3 | 5’-ACCGGCGTCCCTACAATTAC-3’ | 5’-ACCGGCGTCCCTACAATTAC-3’ |
| GAPDH | 5ʹ-GCATCAGCCACTGTTTCAGG-3ʹ | 5ʹ-GCATCAGCCACTGTTTCAGG-3ʹ |
